# Economic impact of biologic utilization patterns in patients with psoriatic arthritis

**DOI:** 10.1007/s10067-017-3636-3

**Published:** 2017-05-04

**Authors:** Sergio Schwartzman, Yunfeng Li, Huanxue Zhou, Jacqueline B. Palmer

**Affiliations:** 1 The Hospital for Special Surgery, 535 E 70th St, New York, NY 10021 USA; 2 Novartis Pharmaceuticals Corporation, East Hanover, NJ USA; 3 KMK Consulting Inc., Morristown, NJ USA

**Keywords:** Above-label dosing, Disease-modifying antirheumatic drug, Dose escalation, Drug costs, Psoriatic arthritis

## Abstract

The aim of the study is to examine the frequency and costs associated with above-label dosing of biologics in patients with psoriatic arthritis (PsA). MarketScan identified adults with ≥1 International Classification of Diseases, Clinical Modification diagnosis for PsA and ≥1 pharmacy claim for biologics of interest between January 1, 2011 and December 31, 2013. The first biologic claim was the index date with a 1-year follow-up period and three additional months to confirm continuous biologic use. Exclusion criteria included switching to a different biologic or diagnosis with another autoimmune disease. During the follow-up period, duration was stratified into three groups: <30, 30–179, and ≥180 days of above-label dosing (>10% of the labeled dose). One-tailed *t* test was conducted to examine the impact of above-label duration on healthcare costs. We identified 4245 PsA patients receiving etanercept (*n* = 2342), adalimumab (*n* = 1788), and golimumab (*n* = 115). Above-label dosing of <30 days (85% adalimumab, 90.4% etanercept, and 95.7% golimumab) and ≥180 days (9.6% adalimumab, 4.1% etanercept, and 2.6% golimumab) was observed. All-cause total healthcare costs for <30 days of above-label use (etanercept $30,625, adalimumab $31,620, and golimumab $37,224), 30–179 days (etanercept $35,602, adalimumab $38,915, and golimumab $64,349), and ≥180 days (etanercept $55,349, adalimumab $54,176, and golimumab $47,993) were reported. Longer above-label duration (30–179 versus <30 days, ≥180 versus 30–179 and ≥180 days) with etanercept or adalimumab was significantly associated with higher mean increased total all-cause healthcare, PsA-specific healthcare, and biologic costs (*p* < 0.05). Above-label use of anti-TNF biologics does occur and is associated with significantly increased healthcare costs.

## Introduction

Psoriatic arthritis (PsA) is a chronic disease which requires aggressive and continuous treatment to manage symptoms and prevent disability [1]. Biologic disease-modifying antirheumatic drugs (bDMARDs, also referred to as biologics) are currently recommended for patients with PsA [2, 3]. A lack of PsA symptom control with initial bDMARD use has been reported to influence decisions to change medications or request additional treatment options [1, 3–7]. Traditionally in a non-responder, a physician may switch the patient from one bDMARD to another bDMARD or change the dose of the therapy being utilized [3–8]. Treat-to-target strategies are also being recommended as a patient-centric approach for managing PsA. Minimal disease activity criteria have been developed to provide targets for tailoring treatments according to patient needs [9, 10]. A recent clinical trial examined a treat-to-target approach and reported statistically significant improvements in both disease activity as well as patient-reported outcomes without any unexpected safety concerns [11].

Recent studies have shown that optimizing bDMARD therapy via off-label dosage for patients with rheumatic diseases is becoming more routine [12–14]. Limited research has been conducted to support long-term health outcomes associated with patterns of bDMARD utilization in the symptom management of PsA [3, 4, 12, 15, 16]. Off-label dosages (i.e., dose escalation or reduction, interrupted treatment) have been shown to occur in clinical practice for the treatment of other inflammatory conditions, such as psoriasis (PsO) and rheumatoid arthritis (RA) [8, 13, 17–20]. Studies have demonstrated that dose escalation with bDMARDs commonly occurs for patients with RA and results in increased cost [13, 18–22]. Off-label dosage has also been examined in patients with PsO, where dose escalation in non-responders generally resulted in increased efficacy with certain bDMARDs (etanercept, adalimumab, infliximab, ustekinumab, and alefacept) [17, 23].

An understanding of the economic implications of real-world medication utilization patterns to manage and control the symptoms of PsA is needed. bDMARDs are reported to be cost-effective for the treatment of moderate-to-severe PsA, mainly due to substantial improvement in decreasing disease activity, preventing radiographic progression, and improving functional status and quality of life [2, 24, 25]. A recent US commercial claims study reported that the annual costs associated with bDMARD treatment for PsA were $$26,916, $27,987, $28,749, and $31,974 (2015 US dollars) for etanercept, golimumab, adalimumab, and infliximab, respectively [26]. Annual direct medical costs for patients with PsA have been reported as $5108 ($22,258) (2012 US dollars, mean [SD]) [27]; however, to date, economic studies for PsA do not provide information on the cost of off-label treatment with patients on biologics and cost-effectiveness of off-label dosing [2, 24, 25]. Due to the long-term nature of treating PsA, the costs of real-world medication utilization of bDMARDs can define treatment options for clinicians and formulary decision makers [28]. This study describes the patient demographics, medication utilization patterns, and associated total healthcare costs among patients with PsA receiving subcutaneous bDMARDs in a real-world setting in the USA.

## Materials and methods

### Data source

A retrospective administrative claims database analysis was conducted using Truven Health Analytics MarketScan^®^ Commercial and Medicare Databases in the USA [29]. The MarketScan Commercial and Medicare Databases provide detailed cost, utilization, and outcome data for healthcare services performed in both inpatient and outpatient settings. Unique enrollee identifiers link medical claims to outpatient prescription drug claims and person-level enrollment data. Database constructs include information on patient demographics (age, gender, employment status, and geographic location), healthcare utilization, costs (payment), and comprehensive prescription drug data [29, 30].

All study data were accessed using techniques compliant with the Health Insurance Portability and Accountability Act of 1996. No identifiable or protected health information was extracted during the course of the study; hence, the study did not require informed consent or institutional review board approval.

### Sample selection and patient population

Adult PsA patients enrolled in the Truven Health MarketScan Commercial and Medicare Supplemental Claims Databases were identified between January 1, 2011 and December 31, 2013 (identification period) [29]. Patients’ first bDMARD claim was the index date, followed by a 1-year follow-up period and an additional 3-month look-forward period to confirm continuous enrollment and biologic use. The study period ended March 31, 2015. Patients included in the study had ≥1 International Classification of Disease, Ninth Revision, Clinical Modification (ICD-9-CM) claim for PsA (ICD-9-CM code 696.0) 1 year before or at the date of first bDMARD use and ≥1 pharmacy claim for etanercept, adalimumab, certolizumab, golimumab, and ustekinumab during the identification period. Intravenous bDMARDs were not evaluated due to limited dosing information in the claims database.

Patients were excluded from the study if they were <18 years of age and had switched to a different bDMARD (including infliximab) during the study period. Patients were also excluded if they had a diagnosis for any of the following diseases: ankylosing spondylitis (ICD-9-CM 720.0), RA (ICD-9-CM 714.x), juvenile idiopathic arthritis (ICD-9-CM 714.3), Crohn’s disease (ICD-9-CM 555.x), ulcerative colitis (ICD-9-CM 556.x), and uveitis (ICD-9-CM 364.0) in order to confirm that the bDMARD treatment was for PsA. Those with human immunodeficiency virus, cancer, and tuberculosis were also excluded to ensure that drug was not discontinued due to a comorbidity.

### Treatment cohorts

bDMARDs of interest were etanercept, adalimumab, certolizumab, golimumab, and ustekinumab. Patients who received certolizumab (*n* = 0) or ustekinumab (*n* = 14) were not included due to small sample size leaving only anti-TNF users in the study since the approvals for these medications were in late 2013. Treatment cohorts were established based on their corresponding index bDMARDs (i.e., etanercept, adalimumab, and golimumab).

### Demographic and baseline patient characteristic variables

Variables including patient demographics, clinical characteristics, and total all-cause healthcare costs (medical and pharmacy) were collected and evaluated for 1 year prior to the index date. Demographic variables of interest were age, gender, insurance type (fee for service (FFS) versus health maintenance organization and geographic region [northeast, north central, south, west, and unknown]). Clinical characteristics collected were concomitant use of non-biologic DMARDs (methotrexate, sulfasalazine, and leflunomide) and PsA-related comorbidities (e.g., PsO [ICD-9-CM 696.x], cardiovascular disease [ICD-9-CM 429.2, 413.x, 410.x, 425.x, 428.x, 430–438], hypertension [ICD-9-CM 401.x], hyperlipidemia [ICD-9-CM 272.x], type 2 diabetes [ICD-9-CM 250.x2], obesity [ICD-9-CM 278.xx], respiratory disease [ICD-9-CM 493.x, 491.x, 492.x, 496.0, 493.2x, 327.2x], gastrointestinal disease [ICD-9-CM 533.x, 564.1, 555.x, 556.x], neurological disorders [ICD-9-CM 356.8, 345.xx, 340.0], liver disease [ICD-9-CM 571.8, 573.3], autoimmune disease [ICD-9-CM 240.0–246.0, 579.0, 250.x1, 710.2, 710.0], depression [ICD-9-CM 300.4, 296.2, 296.3], anxiety [ICD-9-CM 313.0, 300.0x], osteoporosis [ICD-9-CM 733.0], and fibromyalgia [ICD-9-CM 729.1]).

The Quan’s Elixhauser comorbidity index (ECI) score was used to measure the burden of comorbid conditions not directly related to PsA. To differentiate 31 comorbid conditions from complications associated with the disease, ECI utilizes the ICD-9 codes of only the secondary diagnoses unrelated to the primary disease of interest, in this case PsA (i.e., PsA). The mean ECI score and proportion of patients reporting comorbidities for each condition were analyzed [31]. In addition, the Chronic Conditions Warehouse algorithm measured the occurrence of selected PsA-related comorbidities [31–33].

### Outcome measures

The primary outcome for this study was above-label utilization of etanercept, adalimumab, and golimumab during the 1-year follow-up period among patients with PsA. Daily dose for each patient was calculated as total dose of bDMARD for each refill divided by day supply; daily dose was classified into above-label, below-label, or on-label in comparison with labeled dose of each bDMARD. Labeled dosages in PsA for each biologic were as follows: 50 mg once weekly for etanercept [34], 40 mg every other week for adalimumab [35], and 50 mg once a month for golimumab [36]. Within each treatment cohort, utilization included mean days of above-label, below-label, and on-label use (defined as dose >10, <10, and ±10% of the labeled dose, respectively). Duration was stratified into three groups: those with minimal to no above-label use (<30 days), 30–179 days of above-label use, and those with a significant amount of above-label use (≥180 days) during the 1-year follow-up period. Patient demographics and total annual healthcare costs (pre-index and follow-up years) were captured.

### Data analysis

Demographics and comorbidities for 1 year prior were compared for the etanercept, adalimumab, and golimumab cohorts. All continuous variables are presented as mean and standard deviation (SD). All categorical variables are presented as percentages or frequencies. The mean number of days above-label or below-label as well as the number of patients grouped by above-label duration (<30, 30–179, and ≥180 days) were tested for significance between treatment cohorts using the Wilcoxon Mann-Whitney test for continuous variables and the chi-squared test (the Fisher’s exact test was employed when at least 20% of the cells had an expected value less than 5) for categorical/dummy variables.

All costs were converted to 2014 US dollars using the Medical Consumer Price Index. Total all-cause healthcare costs were normalized to annualized costs. A one-tailed *t* test was conducted to examine the impact of the duration of above-label dosing (30–179 versus <30 days, ≥180 versus 30–179 days, and ≥180 versus <30 days) on mean total healthcare costs (all-cause, PsA-specific, biologics, and non-biologics) in the follow-up period. The incremental mean total healthcare cost in the post-index and pre-index periods were compared among above-label dosing groups (30–179 versus <30 days, ≥180 versus 30–179 days, and ≥180 versus <30 days).

## Results

### Baseline demographic characteristics

**Fig. 1:**
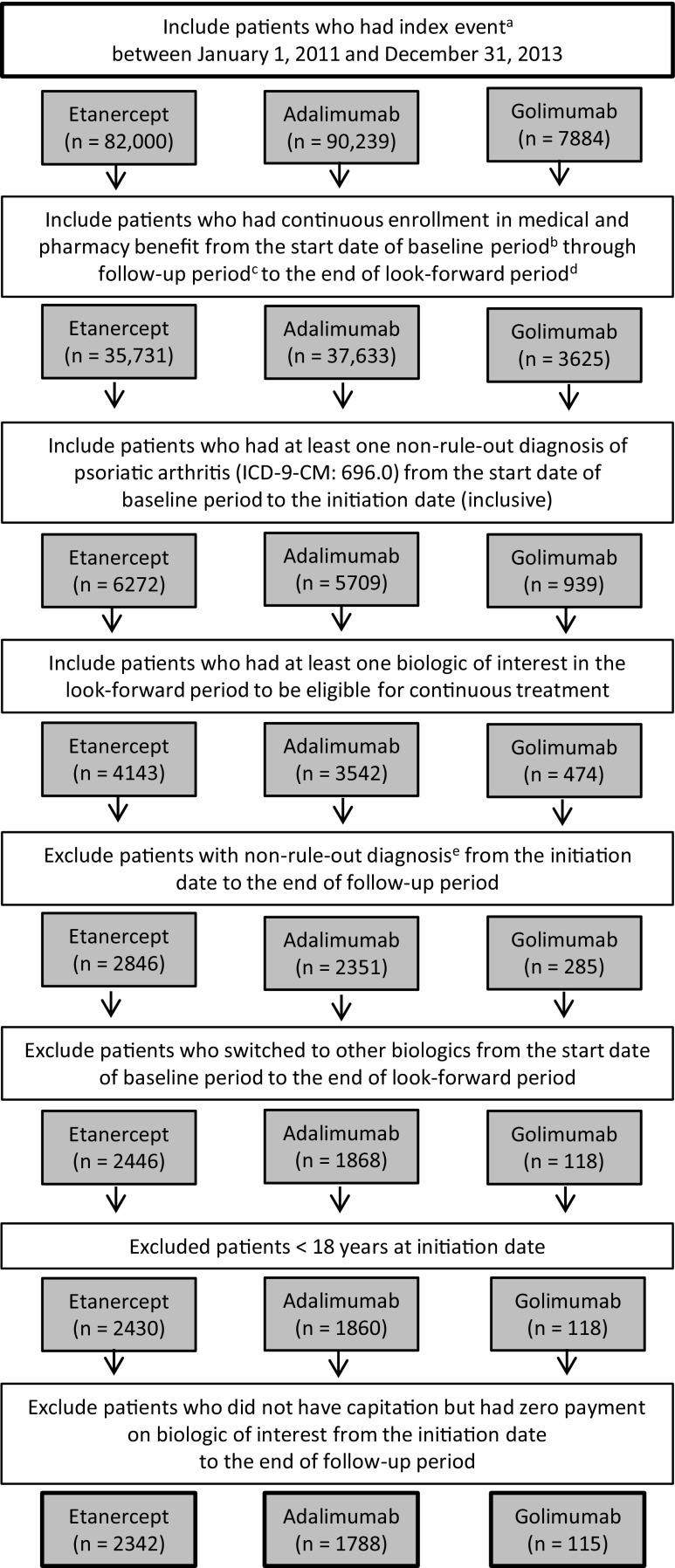
Patient selection chart. ^a^First use of biologic of interest is the index event. ^b^Baseline period is defined as 365 days prior to the index date. ^c^Follow-up period is defined as 365 days after the index date. ^d^Look-forward period is defined as 90 days after the end of the follow-up period. ^e^Non-rule-out diagnoses were ankylosing spondylitis, rheumatoid arthritis, Crohn’s disease, juvenile idiopathic arthritis, ulcerative colitis, uveitis, human immunodeficiency virus, cancer, and tuberculosis. Data source: MarketScan Commercial and Medicare Supplemental databases (http://truvenhealth.com/your-healthcare-focus/analytic-research/marketscan-research-databases). *ICD-9-CM* International Classification of Disease, Ninth Revision, Clinical Modification

The final study population included 4245 PsA patients: etanercept cohort (*n* = 2342), adalimumab cohort (*n* = 1788), and golimumab cohort (*n* = 115) (Fig. 1).

Baseline demographics were similar across treatment groups. The majority of patients were males (etanercept [59.0%], adalimumab [58.6%], and golimumab [52.2%]), with a mean age of approximately 50 years, predominately residing in the southern region of the USA with FFS health insurance. Most patients had at least one PsA-related comorbidity as well as multiple concomitant medications. The majority of PsA patients were treatment experienced, with etanercept showing the highest proportion of patients with prior biologic use (67.0%), followed by golimumab (57.0%) and adalimumab (56.0%) (Table 1).

**Table 1:** Baseline demographic characteristics by etanercept, adalimumab, and golimumab cohorts

	Etanercept cohort (*n* = 2342)	Adalimumab cohort (*n* = 1788)	Golimumab cohort (*n* = 115)
Age (years), mean (SD)	50.7 (10.9)	49.5 (11.3)	48.8 (12.6)
Age group, *n* (%)			
18–24	30 (1.3)	48 (2.7)	6 (5.2)
25–34	145 (6.2)	127 (7.1)	8 (7.0)
35–44	463 (19.8)	396 (22.1)	24 (20.9)
45–54	831 (35.5)	558 (31.2)	37 (32.2)
55–64	694 (29.6)	555 (31.0)	34 (29.6)
65+	179 (7.6)	104 (5.8)	6 (5.2)
Gender, *n* (%)			
Female	960 (41.0)	740 (41.4)	55 (47.8)
United States Geographic Region, *n* (%)			
Northeast	416 (17.8)	243 (13.6)	19 (16.5)
North central	612 (26.1)	430 (24.0)	28 (24.3)
South	796 (34.0)	730 (40.8)	47 (40.9)
West	500 (21.3)	367 (20.5)	18 (15.7)
Unknown	18 (0.8)	18 (1.0)	3 (2.6)
Health insurance			
FFS	1900 (81.1)	1488 (83.2)	102 (88.7)
HMO and POS	408 (17.4)	280 (15.7)	13 (11.3)
Unknown	34 (1.5)	20 (1.1)	0 (0.0)
Medication burden, mean (SD)^a^	7.3 (5.3)	8.1 (5.5)	8.8 (6.0)
Biologic naive, *n* (%)^b^	783 (33.0)	791 (44.0)	49 (43.0)
Biologic experienced, *n* (%)^b^	1559 (67.0)	997 (56.0)	66 (57.0)
Elixhauser comorbidity score, mean (SD)	1.0 (1.2)	1.1 (1.4)	1.2 (1.3)
PsA-related comorbidity, *n* (%)	1752 (74.8)	1380 (77.2)	81 (70.4)
Hypertension	672 (28.7)	502 (28.1)	38 (33.0)
Hyperlipidemia	532 (22.7)	361 (20.2)	21 (18.3)
Osteoporosis	406 (17.3)	330 (18.5)	19 (16.5)
Respiratory disease	195 (8.3)	161 (9.0)	11 (9.6)
Autoimmune disease	190 (8.1)	166 (9.3)	14 (12.2)
Depression	108 (4.6)	74 (4.1)	4 (3.5)
Fibromyalgia	107 (4.6)	100 (5.6)	6 (5.2)
Obesity	101 (4.3)	94 (5.3)	5 (4.3)
Anxiety	96 (4.1)	80 (4.5)	7 (6.1)
Cardiovascular disease	80 (3.4)	55 (3.1)	5 (4.3)
Type 2 diabetes	65 (2.8)	78 (4.4)	4 (3.5)
Liver disease	34 (1.5)	25 (1.4)	1 (0.9)
Gastrointestinal disease	16 (0.7)	20 (1.1)	0 (0.0)
Neurological disorder	10 (0.4)	4 (0.2)	1 (0.9)

*FFS* fee for service, *HMO* health maintenance organization, *n* number, *%* percentage, *PsA* psoriatic arthritis, *POS* point of service, *SD* standard deviation

^a^By Universal System of Classification, excluding the biologics etanercept, adalimumab, and golimumab

^b^Biologic experienced: previous biologic use; biologic naive: no prior biologic use in the 6 months before index date

PSA-related comorbidities were similar across the etanercept (74.8%), adalimumab (77.2%), and golimumab (70.4%) cohorts. Hypertension (etanercept 28.7%, adalimumab 28.1%, and golimumab 33.0%, respectively), hyperlipidemia (etanercept 22.7%, adalimumab 20.2%, and golimumab 18.3%), and osteoporosis (etanercept 17.37%, adalimumab 18.5%, and golimumab 16.5%) presented as the three most common PsA-related comorbidities (Table 1).

### Medication utilization patterns

The mean (SD) number of days of on-label, above-label, and below-label and no use was observed for each of the treatment cohorts by above-label category. Mean (SD) number of days of on-label use (golimumab, 295 [77] days; etanercept, 273 [100] days; and adalimumab, 267 [109] days) and above-label use (golimumab, 12 [57] days; etanercept, 17 [60] days; and adalimumab, 35 [89] days) was reported. Below-label use was not reported for golimumab, whereas etanercept and adalimumab reported a mean (SD) of 4 (30) and 1 (12) days, respectively. The mean (SD) number of days with no use was reported for etanercept (71 [72] days), adalimumab (63 [65] days), and golimumab (59 [61] days), respectively. Proportion of days covered appeared to be consistent across each biologic and for each above-label cohort on average about 0.8 (SD 0.2) (Table 2).

**Table 2:** Medication utilization with above-label dosing of etanercept, adalimumab, and golimumab cohorts

	Etanercept cohort (*N* = 2342)	Etanercept cohort by above-label use category^a^			Adalimumab cohort (*N* = 1788)	Adalimumab cohort by above-label use category^a^			Golimumab cohort (*N* = 115)	Golimumab cohort by above-label use category^a^		
		<30 days (*n* = 2118, 90.4%)	30–179 days (*n* = 129, 5.5%)	≥180 days (*n* = 95, 4.1%)		<30 days (*n* = 1520, 85%)	30–179 days (*n* = 97, 5.4%)	≥180 days (*n* = 171, 9.6%)		<30 days (*n* = 110, 95.7%)	30–179 days (*n* = 2, 1.7%)	≥180 days (*n* = 3, 2.6%)
Number of days above label, mean (SD)	17 (60)	0 (3)	99 (38)	279 (57)	35 (89)	2 (7)	96 (42)	292 (55)	12 (57)	0 (4)	129 (57)	341 (29)
Number of days below label, mean (SD)	4 (30)	4 (32)	1 (6)	0 (0)	1 (12)	1 (12)	0 (0)	0 (4)	0 (0)	0 (0)	0 (0)	0 (0)
Number of days on label, mean (SD)	273 (100)	293 (76)	134 (109)	15 (36)	267 (109)	302 (65)	147 (104)	21 (44)	295 (77)	304 (62)	224 (40)	15 (26)
Number of days no use, mean (SD)	71 (72)	68 (69)	132 (104)	71 (52)	63 (65)	60 (63)	122 (93)	52 (49)	59 (61)	61 (62)	13 (18)	9 (8)
PDC, mean (SD)	0.8 (0.2)	0.8 (0.2)	0.6 (0.3)	0.8 (0.1)	0.8 (0.2)	0.8 (0.2)	0.7 (0.3)	0.9 (0.1)	0.8 (0.2)	0.8 (0.2)	1.0 (0.0)	1.0 (0.0)

*PDC* proportion of days covered, *SD* standard deviation

^a^Above-label use defined as dose >10% of the labeled dose and stratified by <30, 30–179, and ≥180 days during the 1-year follow-up period

Most of the patients in each cohort had minimal to no above-label use (<30 days): 90.4% (etanercept), 85.0% (adalimumab), and 95.7% (golimumab), respectively. About 6.0% of patients in the etanercept cohort, 5.4% of patients in the adalimumab cohort, and 1.7% of patients in the golimumab cohort had 30–179 days of above-label use. The highest above-label dosing category (≥180 days above-label use) was observed for 9.6% of adalimumab, 4.1% of etanercept, and 2.6% of golimumab cohort patients (Table 2).

### Total all-cause annual healthcare costs associated with above-label use

In the 12-month follow-up period, total all-cause annual healthcare costs (in US dollars) were assessed for each biologic cohort by above-label category, and total healthcare costs at <30 days of above-label use for each cohort were as follows: etanercept $30,625, adalimumab $31,620, and golimumab $37,224), 30–179 days (etanercept $35,602, adalimumab $38,915, and golimumab $64,349), and ≥180 days (etanercept $55,349, adalimumab $54,176, and golimumab $47,993). Longer above-label dosing duration with either etanercept or adalimumab was associated with significantly (*p* < 0.05) higher total all-cause healthcare costs, total PsA-specific healthcare costs, and total biologic costs (30–179 versus <30 days, ≥180 versus 30–179 days, and ≥180 versus <30 days) (Table 3).

**Table 3:** Total all-cause healthcare costs associated with above-label use of etanercept, adalimumab, and golimumab cohorts

	Etanercept cohort by above-label use category^a,b^			Adalimumab cohort by above-label use category^a,b^			Golimumab cohort by above-label use category^a,b^		
	<30 days (*n* = 2118)	30–179 days (*n* = 129)	≥180 days (*n* = 95)	<30 days (*n* = 1520)	30–179 days (*n* = 97)	≥180 days (*n* = 171)	<30 days (*n* = 110)	30–179 days (*n* = 2)	≥180 days (*n* = 3)
Total healthcare costs in the follow-up period, mean (SD)^c,d^									
All-cause	$30,625 ($17,928)	$35,602^e^ ($16,600)	$55,359^f,g^ ($17,718)	$31,620 ($13,782)	$38,915^e^ ($30,454)	$54,176^f,g^ ($15,841)	$37,224 ($29,411)	$64,349 ($7927)	$47,993 ($8845)
PsA-specific	$23,246 ($6558)	$27,533^e^ ($8424)	$44,827^f,g^ ($9006)	$24,411 ($6300)	$26,911^e^ ($8899)	$45,289^f,g^ ($10,532)	$26,155 ($6975)	$46,607 ($985)	$44,533 ($7896)
bDMARDs	$22,812 ($6390)	$27,104^e^ ($8467)	$44,282^f,g^ ($8941)	$23,919 ($6178)	$26,331^e^ ($8564)	$44,854^f,g^ ($10,526)	$25,381 ($6325)	$46,019 ($1109)	$44,334 ($7710)
nbDMARDs	$7814 ($16,667)	$8498 ($14,802)	$11,076^g^ ($15,918)	$7701 ($12,760)	$12,584^e^ ($28,389)	$9323^g^ ($11,511)	$11,843 ($30,631)	$18,330 ($6818)	$3658 ($3335)
Incremental total healthcare costs per patient (follow-up costs minus pre-index costs), mean (SD)^c,d^									
All-cause	$10,561 ($19,757)	$16,213^e^ ($20,624)	$25,167^f,g^ ($21,120)	$13,446 ($21,321)	$17,623 ($35,969)	$16,251^g^ ($19,983)	$15,299 ($30,310)	$33,481 ($28,679)	$30,201 ($17,553)
PsA-specific	$10,726 ($11,501)	$16,549^e^ ($16,862)	$23,730^f,g^ ($20,097)	$14,412 ($12,192)	$16,481 ($14,642)	$18,327^g^ ($15,647)	$14,921 ($12,242)	$34,494 ($17,802)	$34,606 ($19,327)
bDMARDs	$10,818 ($11,099)	$17,240^e^ ($15,376)	$23,562^f,g^ ($20,049)	$14,480 ($12,115)	$16,593 ($14,407)	$18,464^g^ ($15,459)	$14,803 ($12,087)	$35,030 ($16,650)	$34,789 ($19,142)
nbDMARDs	$–257 ($16,690)	$–1027 ($15,202)	$1606 ($15,199)	$–1035 ($18,038)	$1030 ($32,444)	$–2213 ($14,192)	$496 ($31,392)	$–1549 ($12,029)	$–4587 ($4482)

*bDMARDs* biologic disease-modifying antirheumatic drugs, *nbDMARDs* non-biologic disease-modifying antirheumatic drugs, *PsA* psoriatic arthritis, *SD* standard deviation

^a^Above-label use defined as dose >10% of the labeled dose and stratified by <30, 30–179, and ≥180 days during the 1-year follow-up period

^b^One-tailed *t* test was conducted to compare the above-label use groups (*p* < 0.05)

^c^Adjusted to 2014 US dollars

^d^All-cause and PsA-related healthcare costs represent medical and prescription drug costs

^e^ *p* < 0.05 for 30–179 versus <30 days

^f^ *p* < 0.05 for ≥180 versus 30–179 days

^g^ *p* < 0.05 for ≥180 versus <30 days

When compared to the prior year, the difference in mean all-cause healthcare costs in each cohort was as follows: etanercept $10,561 (<30 days), $16,213 (30–179 days), and $25,167 (≥180 days); adalimumab $13,446 (<30 days), $17,623 (30–179 days), and $16,251 (≥180 days). Longer above-label dosing duration with etanercept was associated significantly (*p* < 0.01) with increased differences in total all-cause healthcare costs, total PsA-specific healthcare costs, and total biologic costs (30–179 versus <30 days, ≥180 versus 30–179 days, and ≥180 versus <30 days). In addition, adalimumab was also associated with a significantly increased difference in PsA-specific healthcare and biologic costs for ≥180 versus <30 days (*p* < 0.001) (Table 3).

For both analyses above, limited observations were available for the golimumab cohort due to the small sample size (<30 days: golimumab [*n* = 110], 30–179 days: golimumab [*n* = 2], and ≥180 days: golimumab [*n* = 3]), hence hindering the validity of data for statistical evaluation (Table 3).

## Discussion

The current study is the first to describe medication utilization and the economic costs of above-label dosing of anti-TNF biologic therapy for patients with PsA in a real-world setting in the USA. Above-label dosing for 30–179 days was observed in approximately 6% of those receiving etanercept and adalimumab as well as 2% receiving golimumab. Clinically significant above-label dosing of ≥180 days was observed for nearly double the patients receiving adalimumab than etanercept (10 versus 4%, respectively). Furthermore, compared to etanercept, the mean number of days with above-label use was significantly higher for adalimumab and the mean number of days with below-label use was significantly lower in the etanercept group (both *p* = 0.01). Studies in RA populations have also reported more frequent dose escalation among those taking adalimumab versus etanercept [13, 14, 18–20], with two studies reporting a statistically significant difference [13, 20]. This increased utilization in PsA could be due to the different doses used for adalimumab in RA (weekly or every other week approved) versus PsA (every other week) or the varying experience of the treating physician [34, 35].

In this study, higher mean annual total all-cause healthcare costs per patient were associated with increasing duration of above-label anti-TNF dosing (etanercept $30,625 for <30 days versus $55,359 for ≥180 days; adalimumab $31,620 for <30 days versus $54,176 for ≥180 days; golimumab $37,224 for <30 days versus $47,993 for ≥180 days). For both etanercept and adalimumab, increased duration of above-label dosing (30–179 versus <30 days, ≥180 versus 30–179 days, and ≥180 versus <30 days) was significantly associated with higher healthcare costs. Compared to the prior year, the difference in mean all-cause healthcare costs increased in each cohort. Longer duration of above-label etanercept use was significantly associated with greater differences in healthcare costs (30–179 versus <30 days, ≥180 versus 30–179 days, and ≥180 versus <30 days; all *p* < 0.01), whereas in the adalimumab cohort, higher healthcare costs were only significant for the ≥180 versus <30-day comparison (*p* < 0.05). Limited observations were available for the golimumab cohort due to the small sample size.

In the USA, the direct annual healthcare costs for PsA are estimated to be as high as $1.9 billion. Indirect costs associated with PsA account for 52 to 72% of the total annual costs. Both direct and indirect costs associated with PsA increase with worsening physical function and disease activity [34]. Our study estimated that the total annual anti-TNF costs for those with less than 30 days of above-label dosing were approximately $22,812 for etanercept, $23,919 for adalimumab, and $25,381 for golimumab. These findings are aligned with another US commercial claims database analysis, which reviewed anti-TNF biologic therapy across different indications and reported similar annual treatment costs as reported in this study for PsA across the anti-TNF biologics, etanercept ($26,916), golimumab ($27,987), and adalimumab ($28,749) [26]. The current study was the first to report that the total annual PsA, anti-TNF biologic-related, and PsA-specific healthcare costs significantly increased with the duration of above-label dosing. This study also evaluated the cost of non-biologic DMARDs which are often added to therapy and could have cost implications, especially patients with above-label use.

When dealing with a lack of treatment response, it is important to understand and consider the cost implications of off-label utilization in the real-world setting [8]. The current study is the first to provide an estimated total annual all-cause healthcare cost of $55,349 (etanercept), $54,176 (adalimumab), and $47,993 (golimumab) US dollars for above-label use (≥180 days) in PsA. The impact of these costs among patients with ≥180 days of above-label use could equate to $2,040,885 (etanercept), $3,345,444 (adalimumab), and $45,012 (golimumab), respectively. Similar cost estimates for above-label use of etanercept and adalimumab have been reported in a real-world population of RA patients, with a range of $20,000 to $23,000 US dollars [13, 14].

The economic impact of above-label prescribing should be taken into consideration along with the benefits and safety risks in clinical practice [17]. Therapy modifications may require an individualized approach to account for factors such as disease severity, quality of life, and comorbidities [17, 23]. In studies of PsO, dose escalation—primarily an increase in dosing frequency—is performed when patients fail to respond, or only partially respond, to the standard dose [17]. Indeed, dose escalation with etanercept (50 mg twice weekly) and adalimuab (40 mg weekly) has been associated with greater efficacy than standard dosing [17]. Among patients with rheumatoid arthritis, increased dosing frequency generally occurs 4–9 months after treatment initiation [37].

For patients with PsA, dose escalation may reflect a partial response to the standard treatment dose or a flare-up of symptoms or disease progression. Alternatively, it may suggest that physicians are unaware of the treatment guidelines for PsA, which recommend a switch in biologics for patients who do not achieve minimum disease activity after 3–6 months of treatment [15, 38]. The guidelines do not discuss the practice of above-label dosing but do emphasize the importance of evaluating risks of treatment, with regard to overall safety and effects on comorbidities [15, 38]. An understanding of the reasons for above-label dosing and the impact on efficacy and safety of these regimens through further studies is critical for effective decision-making and care of patients with PsA.

This claims-based analysis study has limitations that should be noted. Patient information that may influence dose escalation such as disease severity and concurrent treatments for PsA were not captured, and reasons for above-label dosing were not available. Administrative claims data were not collected for research purposes, and diagnoses on claims may have been coded incorrectly or not coded at all, thereby potentially introducing measurement error with respect to ICD-9-CM-based variables. The current study was limited to individuals with commercial health coverage; therefore, findings may not be generalizable to people with Medicaid, Medicare, other insurance, or no insurance. Using a retrospective database approach limits the study to those who are clinically diagnosed and receive medications through their insurance. Since patients were not randomized to the different treatments, there may be some uncontrolled biases that could affect treatment outcomes. Provider bias for above-label dosing to control symptoms may also have influenced the utilization patterns observed among those receiving bDMARDs. Overall, these limitations are typical of any claims-based analysis and do not impede the conclusions drawn regarding the real-world utilization and costs associated with above-label doses of bDMARDs for patients with PsA in the USA.

## Conclusion

In conclusion, this retrospective, real-world study from a large US claims database observed above-label dosing of almost all anti-TNFs approved for PsA, etanercept, adalimumab, and golimumab, with the exclusion of infliximab. Significantly higher healthcare costs per patient were associated with a longer duration of above-label dosing. Even minimal above-label doses of etanercept and adalimumab were shown to be associated with significantly increased healthcare costs. While the majority of patients in the study were observed with <30 days of above-label dosing, a subset of patient had dose escalation which may suggest an inadequacy of standard anti-TNF dosing regimens for PsA populations with prior biologic experience. More research to understand reasons for above-label prescribing in a real-world setting could aid physicians and decision makers in defining ideal treatment regimens.
